# Quality of life improvement in Latinas receiving combined substance use disorders and trauma-specific treatment: a cohort evaluation report

**DOI:** 10.1186/s12955-017-0667-z

**Published:** 2017-05-02

**Authors:** Miguel A. Cruz-Feliciano, Christine Miranda-Díaz, Diana M. Fernández-Santos, Darice Orobitg-Brenes, Robert F. Hunter-Mellado, Ibis S. Carrión-González

**Affiliations:** 1 Institute of Research, Education and Services in Addiction, Universidad Central del Caribe School of Medicine, PO Box 60327, Bayamon, PR 00960-6032 Puerto Rico; 2 Internal Medicine Department, Universidad Central del Caribe School of Medicine, PO Box 60327, Bayamon, PR 00960-6032 Puerto Rico

**Keywords:** Quality of life, Women, Substance use disorders, Latinas

## Abstract

**Background:**

This study evaluates the benefits of integrating behavioral health and trauma services for Latinas with a history drug use. Changes in quality of life (QOL) domains were documented after participation in a manualized intervention in a cohort of Latinas.

**Methods:**

Participants were part of a prospective cohort study of 136 Latinas with co-occurring disorders (COD) who may have experienced trauma and receiving services in our outpatient treatment facility in Bayamón, Puerto Rico. The WHOQOL-BREF Spanish version was used to score physical, psychological, social, and environmental QOL domains, at intake and after six months. Sociodemographic variables, alcohol, drug use, mental health disorders, and severity of substance use disorders (as defined by the DSM-5) were also tabulated. Descriptive statistics and paired *t* test or the Wilcoxon signed-rank test were computed for comparison.

**Results:**

A median age of 39 years was seen and with 76% high school education or higher degree. The majority were unemployed (95.9%). A diagnosis of severe cocaine use (51.4%) was present and almost half (49.5%) had three or more DSM-5 diagnoses. Mean QOL scores were higher at six months with statistically significant differences in each domain. Women with neurodevelopmental disorders and schizophrenia yielded higher mean QOL scores for each domain at six months except for the social domain. Women with polydrug use and women who reported exposure to trauma and depressive disorder experienced statistically significant increments in the physical, psychological and social domains in comparison to counterpart women.

**Conclusions:**

Significant and positive changes in QOL were found in each domain. Latinas who reported traumatic events had lower scores in the physical and psychological QOL domains. There was a high prevalence of diminished physical and mental functioning in Latinas with COD. The exposure to trauma and the lack of social support negatively affect treatment access and retention for Latinas.

## Background

Behavioral health, including mental health, is a major concern. As of 2014, an estimated of 21.5 million persons aged 12 or older had substance use disorders (SUDs) and about 1.0% of the adult population had co-occurring disorders (COD) [1]. The quality of life (QOL) is a significant indicator of wellness among individuals and has been studied as a relevant clinical construct in the provision of care for substance use, and recovery services. The negative effects of SUDs in the multiple domains of QOL have been documented [2–7]. In addition, co-occurring mental health disorders and sociodemographic factors including gender, age, and educational attainment are mediating variables which influence QOL in individuals with substance use disorders (SUD) [5, 8, 9]. Published studies suggest that women in treatment for alcohol use disorder with co-occurring depression had lower QOL than women without depressive symptoms [10–13]. Gender disparities may exist in women who engage in SUD treatment services in terms of quality of health status, social context, and environmental conditions (e.g., housing, safety, and security) in the US [14]. In Puerto Rico, greater disparities in treatment provision and access to services were found in women with SUD seeking services as compared to men [15]. The literature suggests that Puerto Rican women with SUD tend to report a lower perception of well-being, higher prevalence of chronic diseases, depression symptoms, and lower social support than women with SUD in the US [15]. For women with SUD in the US, lower scores in the environmental QOL over time and a decrease in the social QOL score one month after treatment have been reported [14]. Other authors have presented data suggesting that recent alcohol or drug use, being in treatment, and the number of sessions attended were not significantly associated with improvements in QOL among women with SUD [14].

SUD and exposure to trauma-related events have been shown to negatively influence treatment outcomes [16, 17]. Women with SUD seeking substance use treatment have greater psychological distress, mental health problems, and have experienced traumatic events including sexual and physical assault as compared to men [18–25]. Trauma has been significantly correlated with physical and psychological QOL domains in previous studies but is not well understood in Latinas [14, 26, 27]. A recent study found an association between depression, substance use, and being a victim of sexual abuse during childhood and adulthood in Latinas [28]. Other studies have shown that women who use substances are significantly more likely to experience intimate partner violence (IPV) which is associated with increased drug and alcohol use [29–37], making substance use both a risk factor for and a consequence of IPV. The development of posttraumatic stress disorders (PTSD) and related symptoms that result from exposure to trauma should be examined as a factor that negatively affects treatment outcomes in populations with SUD and co-occurring depression [17].

Successful recovery from SUD has been associated with how individuals appraise and cope with stressful life events. Studies indicate that there is an association between higher perceived social support and better quality of life in the populations with SUD [38]. Women with SUD tend to perceive less social support than men, which has been shown to negatively affect treatment access and retention [39]. Moreover, higher levels of social support were predictors of higher levels in all QOL domains [38, 40]. Previous researchers have stressed the need of integrated treatment models that address SUD, QOL, and trauma in women [16, 41, 42]. In Puerto Rico, treatment services for women with substance use disorders and trauma have been offered in a fragmented manner. The literature indicates that gender-specific, culturally responsive, comprehensive services may improve retention and outcomes for Latinas [43]. *Proyecto Mujer* (Project Woman) is an integrated trauma and substance use treatment service for Latinas with COD and who are at risk for HIV/AIDS and hepatitis infection. *Proyecto Mujer* is framed in the motivational interviewing (MI) and the transtheoretical model of change (TMC) and includes core components of Seeking Safety (SS) [44], and RESPECT (Intervention developed by Kamb et al. under the Project RESPECT study) [45, 46] evidence-based practices (EBPs). The MI and TMC approaches were incorporated to improve women’s outcomes. SS focuses on safety from substance use and IPV as its main clinical area whereas Project RESPECT Enhanced Counseling was included to reduce HIV risk behaviors commonly observed in populations with SUDs.

This study aims to evaluate the benefits of an integrated behavioral health and trauma-specific services for Latinas with substance use disorders and mental illness. We describe changes in each QOL domain after enrollment in *Proyecto Mujer* as compared to six months later. We also identify the mediating role of sociodemographic variables and DSM-5 diagnosis criteria [47] as correlates of QOL in Latinas with COD.

## Methods

### Setting and procedures

Participants were part of a prospective cohort study of 136 Latinas with co-occurring disorders (COD) who may have experienced trauma and receiving services in our outpatient treatment facility in Bayamón, Puerto Rico. After the initial screening, participants signed an informed consent. Face-to-face interviews were conducted at intake and six months from January 2014 to September 2015. All interviews were conducted by trained case management specialists and the clinical assessment was performed by a licensed clinical psychologist. The intake and follow-up interview, as well as the clinical assessment time duration, were approximately 1 h each. Strictly voluntary urine test for the identification of psychoactive substances, OraQuick Advance HIV-1/2 Antibody test and counseling, and Hepatitis B and C test were offered to the study’s participants. Positive test results were sent to the laboratory for confirmation and for treatment referral. Participants received $20.00 incentive for the follow-up assessment completion. The study was reviewed and approved by the Universidad Central del Caribe Institutional Review Board (IRB# 2013–24).

### Participants

A total of 136 women receiving services at the clinic during the period of 14 months represented the sample of this investigation. The power of the sample was determined to be robust for statistical significance for the majority of the groups investigated. The power of the sample size for each domain is presented in Table 3. Follow-up measure was conducted at 6 months and was completed by 107 women; representing a 79% retention rate. Eligible participants were (a) women with SUD and/or COD who are living with HIV or at risk for HIV at the time of intake, (b) between 18–64 years of age, (c) with positive 9-panel drug test or visible fresh needle tracks, (d) living in the metropolitan area of Puerto Rico; and (e) with limited or no health insurance coverage.

### Instruments and measures

Data collected included sociodemographic information (age, educational attainment, race/ethnicity, employment status, monthly income, marital status, and health insurance), alcohol and drug use, mental health disorders, and severity of SUD (as defined by the DSM-5).

The WHOQOL-BREF Spanish version was used to measure physical, psychological, social, and environmental QOL domains scores, at intake and six months after intake. The areas of QOL represent categories of functioning within each domain. The physical domain refers to elements of activities of daily living, energy, mobility, pain, sleep, rest and work capability. The psychological domain for QOL is associated with body image, appearance, negative and positive feeling, self-esteem, memory, and concentration. The social domain describes personal relationships, social support, and sexual activity. The environmental denotes financial resources, freedom, security, safety, opportunities for recreation and leisure. The WHOQOL-BREF is a 26-item abbreviated form of the WHOQOL-100 developed by the World Health Organization [48]. The WHOQOL-BREF guidelines were followed to calculate raw domain scores and transform them to the 0–100 scale [48]. Normative means scores for each QOL domain were calculated for the general population by other investigators [49, 50]. For the purposes of our study, we have used the following normative mean scores as comparison: 75.1 (SD = 13)/ 74 (SD =16) for the environmental domain, 73.5 (SD = 18.1)/ 77 (SD = 17) for the physical domain, 69 (SD = 18)/ 71.5 (SD = 18.2) for the social domain, and 69 (SD =16)/ 70.6 (SD = 14) for the psychological domain [49, 50].

### Treatment intervention

**Fig. 1:**
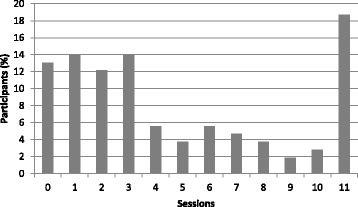
Distribution of session completed by participants (*n* = 107). Note. Average number of sessions completed by each participant

*Proyecto Mujer* treatment intervention consists of 11 sessions delivered within 6 months that includes core components of two EPBs; however, treatment in some instances was completed in an 8-months period. The average number of sessions completed was three; delivered in an individual format (See Fig. 1). SS is a widely used EBP that addresses cognitive, behavioral, interpersonal, and case management issues in the integrated treatment of post-traumatic stress disorder (PTSD) and substance use [51–55]. SS is designed to be flexible in order to adapt to the client’s needs, clinician’s preferences, and a variety of topics [44]. The RESPECT Enhanced Counseling is an EBP that has been shown to provide good evidence to change HIV/STD risk behaviors [45]. RESPECT Enhanced Counseling is a one-on-one client focused HIV/STD prevention intervention that includes four interactive sessions. This EBP also helps participants build a short and long-term HIV risk reduction plan.

SS and RESPECT Enhanced Counseling sessions were alternated and combined starting with the first SS treatment topic (“Introduction to treatment” / Case management) and the first RESPECT Enhanced Counseling session (“HIV personal risk factors and barriers to risk reduction”/Counseling) to engage participants early in treatment. *Proyecto Mujer* includes five SS treatment topics: Safety (combination), When Substances Control You (cognitive), Coping with Triggers (behavioral), Setting Boundaries in Relationships (interpersonal) and Getting Others to Support Your Recovery (interpersonal). These topics were selected based on the previous experiences with this population and the length of the intervention. RESPECT Enhanced Counseling focused on condom use attitudes, social norms, support for condom use, and condom use self-efficacy [56]. *Proyecto Mujer* was delivered by trained case managers, substance use counselors, and a licensed clinical psychologist. To enhance the fidelity of the intervention, sessions were audiotaped and discussed during clinical supervision and group discussion.

Previous research has established the effectiveness of combining MI and TMC in substance use disorders treatment programs [57]. *Proyecto Mujer* used this combined framework to support clients’ motivation for change based on their stage of readiness. MI is a clinical approach applied to substance use in which the clinician enhances client motivation for change through the development of a client-centered therapeutic relationship [58]. The TMC assess patients’ readiness to adopt health-promoting behaviors through five stages of change including precontemplation, contemplation, preparation, action, and maintenance [59, 60].

### Data analysis

Descriptive statistics were used to summarize the characteristics of the participants of the study: frequencies and percentages for categorical variables and central tendency (mean/median) and dispersion measures (SD) for continuous variables. To account for the distribution of continuous variables in the dataset, a normality diagnostic test was performed using the Shapiro-Francia estimator. Independent variables were sociodemographic variables (age, marital status, and educational level) substance use disorders, mental health disorders (personality, bipolar, depression, and anxiety), and trauma. Variables were analyzed using a before/after approach with the ordinary paired *t* test. The significance level (α) was set to ≤ 0.05. Post-hoc power (1 - β) analyses were also conducted and reported. Statistical analyses were performed by IBM Statistical Package for Social Sciences v.20.0 for Windows [61] and G*Power 3.1.3 [62]. Women with neurodevelopmental disorders and schizophrenia (*n* = 23) were excluded from the paired *t*-test analysis due to cognitive impairment [14] and adaptation to the current treatment intervention. In this group, statistically significant differences in treatment variables at six months were not found.

## Results

Table 1 shows the substance use profile, mental health screening, HIV and hepatitis clinical status at intake. Participants on average were 39 years old, 98% identified themselves as Latinas, 76% completed a high school education or a higher degree, and 95.5% were unemployed. Most women (85%) reported being single. At intake, 38.5% reported regular health status. At intake, about 73% of women reported previous exposure to traumatic events.

**Table 1:** Intake and follow-up substance use disorders, mental health disorders, and clinical variables (*n* = 107)

Variable	Intake		Six months	
	*n*	%	*n*	%
SUD ^a^				
Alcohol	47	43.9	31	29.0
Cocaine	34	31.8	17	15.9
Heroin	13	12.1	19	17.8
Cannabis	30	28.0	5	4.7
Benzodiazepine	19	17.8	12	11.2
Tranquilizer	7	6.5	2	1.9
Mental Health Screening ^a^				
Neurodevelopment	18	16.8	--	--
Schizophrenia	6	5.6	--	--
Bipolar	21	19.6	--	--
Depression	41	38.3	--	--
Anxiety	13	12.1	--	--
Trauma	78	72.9	--	--
Disruptive	11	10.3	--	--
Substance use	99	92.5	--	--
Personality	20	18.7	--	--
Suicidal Behavior	8	7.5	--	--
Clinical variables ^a^				
HIV +	16	15.0	--	--
HBV +	3	2.8	--	--
HCV +	11	10.3	--	--

*SUD* Substance use disorders

^a^ Non-mutually exclusive

Table 2 shows substance use disorders as defined by DSM-5 diagnostic criteria at intake. Most women were diagnosed with severe cocaine use (51.4%) followed by alcohol use disorder (29.0%) and opioid use disorder (26.2%). Almost half (49.5%) had three or more DSM-5 diagnoses.

**Table 2:** Substance-related DSM-5 diagnostic criteria at intake (*n* = 107)

DSM-5 diagnostic criteria ^a^	No diagnosis		Mild		Moderate		Severe	
	*n*	%	*n*	%	*n*	%	*n*	%
Alcohol	42	39.3	12	11.2	22	20.6	31	29.0
Cocaine	35	32.7	7	6.5	10	9.3	55	51.4
Cannabis	38	35.5	17	15.9	26	24.3	26	24.3
Opioid	74	69.2	1	0.9	4	3.7	28	26.2

^a^ Non-mutually exclusive

Cronbach's alpha coefficient was applied to examine the internal consistency of QOL domains. We obtained a moderate to high internal consistency for each QOL domains; physical 0.736, psychological 0.792, social 0.651, and environmental 0.811. Table 3 shows the changes in QOL by domain at intake and at six months for the sample (*n* = 107), for women without neurodevelopmental disorders and schizophrenia (*n* = 84), and for women with neurodevelopmental disorders and schizophrenia (*n* = 23). The mean QOL scores were higher at six months with statistically significant differences in each domain (*p* ≤ 0.05): physical domain (mean difference = -4.5 and mean difference = -5.0), psychological domain (mean difference = -6.3 and mean difference = -6.2), social domain (mean difference = -6.9 and mean difference = -9.4), and environmental domain (mean difference = -4.8 and mean difference = -6.1) for all women in the sample and for women without neurodevelopmental disorders and schizophrenia. For women with neurodevelopmental disorders and schizophrenia, the mean QOL scores for each domain were higher at six months except for the social domain: physical domain (mean difference = -2.2), psychological domain (mean difference = -6.0), social domain (mean difference = 2.2), and environmental domain (mean difference = -0.1) but did not reach statistical significance.

**Table 3:** QOL mean scores at intake and follow-up for different populations

QOL Domain	Intake	Six months	*p*-value ^a^	ES^ ^b^	1 - β ^c^
	Mean (SD)	Mean (SD)			
All women (*n* = 107)					
Physical	57.3 (19.8)	61.8 (20.7)	0.015	0.239	0.69
Psychological	59.9 (20.8)	66.2 (21.5)	0.002	0.313	0.89
Social	58.9 (24.1)	65.8 (26.4)	0.008	0.262	0.77
Environmental	55.8 (18.9)	60.6 (19.7)	0.009	0.257	0.75
Women without neurodevelopmental disorders and schizophrenia (*n* = 84)					
Physical	58.8 (19.3)	63.8 (19.0)	0.012	0.280	0.72
Psychological	61.3 (20.1)	67.5 (21.4)	0.007	0.302	0.78
Social	59.4 (24.2)	68.8 (24.3)	0.001	0.379	0.93
Environmental	56.8 (18.5)	62.9 (18.8)	0.002	0.342	0.87
Women with neurodevelopmental disorders and schizophrenia (*n* = 23)					
Physical	52.1 (21.4)	54.3 (25.2)	0.620	0.105	0.08
Psychological	55.2 (23.2)	61.2 (21.7)	0.097	0.361	0.38
Social	57.1 (24.3)	54.9 (31.4)	0.734	0.072	0.06
Environmental	52.0 (20.1)	52.1 (20.8)	0.977	0.006	0.05

^a^ *p*-value from paired *t*-test analysis

^b^ ES = Effect size. ES^ the correlation between measures was considered in the calculation (Cohen’s method)

^c^ Power obtained from the post-hoc analysis

Changes in QOL by domain at intake and at six months by selected sociodemographic characteristics are presented in Table 4. Being single (mean difference = -12.1) or married (mean difference = -9.6) and higher educational attainment (mean difference = -4.2) were all associated with statistically significant increments in the physical domain score (*p* ≤ 0.05). In the psychological domain, having an education equal or less than high school yielded statistically significant differences (mean difference = -13.8). Significant increments were also observed in the social domain (*p* ≤ 0.05) in young or middle-aged adult (mean difference = -9.7; mean difference = -11.5), never married (mean difference = -10.0), and higher educational attainment (mean difference = -10.3). In the environmental QOL domain, being middle-aged (mean difference = -8.3), never married (mean difference = -6.7), and with high or low educational attainment (mean difference = -8.0, mean difference = -5.6) yielded statistical significant differences.

**Table 4:** Change in QOL domains by sociodemographic characteristics (*n* = 86)

	Physical	Psychological	Social	Environmental	Physical	Psychological	Social	Environmental
	Mean (SD)	Mean (SD)	Mean (SD)	Mean (SD)	Mean (SD)	Mean (SD)	Mean (SD)	Mean (SD)
Age								
18-34	57.8 (19.5)	57.7 (21.0)	57.9 (22.3)	56.1 (20.3)	65.1 (15.0)	64.8 (19.1)	*67.6 (23.8)	61.0 (17.0)
35–55	57.4 (19.9)	63.5 (19.0)	59.5 (25.3)	56.2 (18.1)	60.7 (21.7)	67.0 (23.5)	*71.0 (24.8)	*62.9 (20.3)
56–64	69.7 (13.5)	66.0 (22.6)	57.9 (21.8)	67.1 (6.0)	75.1 (8.7)	80.4 (11.6)	70.6 (23.8)	74.3 (13.3)
Marital status								
Single	51.4 (21.4)	60.5 (20.3)	65.5 (23.6)	54.7 (13.6)	*63.5 (19.7)	69.5 (20.2)	73.0 (24.1)	59.3 (16.4)
Married	59.7 (18.2)	58.9 (20.6)	69.6 (26.2)	60.0 (21.6)	*69.3 (16.5)	67.9 (20.3)	78.5 (20.4)	67.7 (21.4)
Never married	60.5 (18.8)	62.1 (20.2)	54.9 (23.0)	56.5 (19.0)	62.4 (19.5)	66.9 (22.3)	*64.9 (24.7)	*62.5 (18.8)
Education								
Less than 12 grade	56.4 (18.1)	58.2 (19.6)	63.7 (24.8)	55.1 (17.4)	65.4 (17.7)	*72.0 (15.6)	69.2 (25.2)	*63.1 (18.2)
HS or higher	59.3 (19.6)	62.0 (20.2)	58.4 (24.1)	57.2 (18.9)	*63.5 (19.4)	66.5 (22.5)	**68.7 (24.2)	*62.8 (19.1)

*p*-value obtained from paired *t*-test. *p < 0.05; **p < 0.01

Changes in QOL by domain by clinical variables are presented in Table 5. Polydrug use was all associated with statistically significant increments in the physical, psychological and social domains (*p* ≤ 0.05). Significant increments (*p* ≤ 0.05) in each domain among those who reported exposure to trauma and depressive disorder were also observed. Those with an anxiety disorder also experience significant increments in the social and environmental domains (*p* ≤ 0.05).

**Table 5:** Change in QOL domains by type of use, history of trauma, and mental health disorders as defined by DSM-5 (*n* = 86)

Variable	Intake				Six-months			
	Physical	Psychological	Social	Environmental	Physical	Psychological	Social	Environmental
	Mean (SD)	Mean (SD)	Mean (SD)	Mean (SD)	Mean (SD)	Mean (SD)	Mean (SD)	Mean (SD)
Type of user								
1 drug	51.4 (19.5)	56.1 (17.2)	41.1 (21.5)	51.3 (21.5)	52.9 (21.6)	63.8 (17.7)	*58.6 (22.4)	*(62.9 (19.9)
Polydrug	61.4 (19.4)	63.7 (20.5)	64.5 (22.3)	59.7 (17.1)	*67.4 (17.0)	*69.8 (21.5)	*72.6 (23.2)	64.1 (17.4)
History of trauma	59.9 (19.3)	63.3 (20.4)	59.9 (21.7)	59.1 (18.2)	*64.9 (18.8)	*70.3 (20.2)	*69.4 (23.3)	*64.5 (17.0)
Bipolar disorder	61.7 (20.4)	61.7 (19.0)	59.1 (22.0)	63.9 (18.8)	59.5 (22.6)	65.7 (24.6)	66.3 (28.3)	65.2 (19.6)
Personality disorder	54.7 (18.5)	59.1 (20.6)	55.0 (27.3)	52.6 (17.3)	59.4 (17.9)	64.3 (21.3)	63.5 (24.9)	56.7 (17.7)
Depressive disorder	58.5 (19.9)	63.3 (19.7)	61.6 (25.3)	56.9 (17.8)	*67.0 (17.8)	*71.7 (20.9)	*72.3 (21.8)	*64.4 (16.6)
Anxiety disorder	53.8 (19.7)	55.8 (23.9)	53.6 (20.1)	55.7 (18.7)	57.0 (19.2)	66.8 (12.3)	*72.3 (18.5)	*63.6 (18.0)

*p*-value obtained from paired *t*-test. *p < 0.05

## Discussion

Mean scores within QOL domains remained significantly lower in our sample as compared with mean scores for women in treatment centers in the US and the general population [14, 49, 50]. Our study confirms previous research that suggests that women with COD obtain lower mean QOL scores than men [10–13]. Similar to a study conducted in the US, women reported lower scores in the environmental domain [14]. A study found that being older was associated with low physical quality of life; however, we did not found significant increments in this domain by age [14]. We confirmed that women reporting traumatic events had lower scores in the physical and psychological QOL domains [14]. As previous research, depressive and anxiety disorders were associated with lower QOL mean scores [13].

Positive changes in QOL were found in each domain. Previous research has emphasized that participation in substance use treatment services did not predict improvements in quality of life [14]. In our study, women’s readiness for change might explain this improvement. *Proyecto Mujer* includes an MI approach which has been shown to be an effective therapeutic tool to influence clients’ behavioral change [58]. Moreover, previous research has suggested that quality of the client-therapist relationship or therapeutic alliance predict treatment outcomes [63, 64]. Other non-specific factors that should be considered are the therapist’s empathy and client-therapist goal consensus and collaboration which have found to support client autonomy and engagement in the therapeutic process [65]. Further research is needed to address the influence of these factors in the overall QOL in the SUD populations.

QOL has been recognized as an outcome indicator of mental health services. This study aimed to explore the benefits of integrating behavioral health and trauma services for Latinas and to describe changes in QOL in each domain after enrollment in *Proyecto Mujer*. Findings indicate positive changes in QOL domains over the six-month period; however, changes in QOL for persons with neurodevelopmental disorder and schizophrenia were not significant. Factors that influence QOL changes for schizophrenia patients include distress factor including severity of symptoms; coping factors such as coping styles, and social support; and personality related factors which include self-efficacy, self-esteem, and emotional distress (with negative loading) [66]. Our findings confirmed previous recommendations for planning combined substance use and trauma-specific services according to the severity of substance use disorders and the co-occurring mental health disorders.

This study has some methodological limitations. Women seeking treatment for SUDs were voluntary participants receiving treatment; generalization to women with SUDs out of treatment is not possible. Definite causal association between treatment and measures improvement cannot be established in this study. Cross-comparison with previous studies is difficult due to the lack of uniformity used to measure QOL. Most studies have measured changes in health-related quality of life rather than changes in the overall QOL which is a critical component for women using substances. The positive improvements in QOL domains yielded in this study should be measured for an extended period to determine if the effects of the intervention remain.

## Conclusions

Results of this study highlight the profile of Latinas with COD seeking treatment for SUDs. The exposure to trauma and the lack of social support negatively affect treatment access and retention for Latinas. Additional studies are needed to examine if *Proyecto Mujer* might serve to further increase the likelihood of better treatment outcomes in women with COD.

## Abbreviations

COD: Co-occurring disorders
EBPs: Evidence-based practices
IPV: Intimate partner violence
MI: Motivational interviewing
PTSD: Post-traumatic stress disorder
QOL: Quality of life
SS: Seeking safety
SUD: Substance use disorders
TMC: Transtheoretical model of change
